# Effect of Long-Term Omega 3 Polyunsaturated Fatty Acid Supplementation with or without Multidomain Lifestyle Intervention on Muscle Strength in Older Adults: Secondary Analysis of the Multidomain Alzheimer Preventive Trial (MAPT)

**DOI:** 10.3390/nu11081931

**Published:** 2019-08-16

**Authors:** Yves Rolland, Philipe de Souto Barreto, Mathieu Maltais, Sophie Guyonnet, Christelle Cantet, Sandrine Andrieu, Bruno Vellas

**Affiliations:** 1 Gérontopôle de Toulouse, Institut du Vieillissement, Centre Hospitalo-Universitaire de Toulouse (CHU Toulouse), F-31000 Toulouse, France; 2 UMR INSERM 1027, University of Toulouse III, F-31000 Toulouse, France

**Keywords:** ω3-PUFA, muscle strength, sarcopenia, physical performances

## Abstract

Background: The benefits of long-term omega 3 polyunsaturated fatty acid (ω3-PUFA) supplementation on muscle strength in older adults remains to be investigated. Objectives: We assessed the effect of ω3-PUFA supplementation and a multidomain (physical activity, cognitive training, and nutritional advice), alone or in combination, compared with placebo, on muscle strength. We also hypothesized that ω3-PUFA supplementation resulted in additional benefit in participants with a low docosahexaenoic acid (DHA)/eicosapentaenoic acid (EPA) erythrocyte level at baseline and high adherence to the multidomain intervention sessions. Design: We performed secondary analyses of the Multidomain Alzheimer Preventive Trial (MAPT), a 3-year, multicenter, randomized, placebo-controlled trial with four parallel groups. Participants were non-demented, aged 70 years or older. They were recruited in 13 memory clinics in France and Monaco between 30 May 2008 and 24 February 2011. Participants were randomly assigned to either ω3-PUFA alone (two capsules a day providing a total daily dose of 800 mg DHA and 225 mg EPA), ω3-PUFA plus the multidomain intervention (43 group sessions integrating advice for physical activity (PA), and nutrition, cognitive training, and three preventive consultations), the multidomain intervention plus placebo, or placebo alone. Our primary outcome was the change from baseline to 36 months of the muscle strength assessed with the repeated chair stand test and handgrip strength. Results: A total of 1680 participants (75.34 years ± 4.42) were randomized. In the modified intention-to-treat population (*n* = 1679), no significant differences at 3-year follow-up were observed in the repeated chair stand test score between any of the three intervention groups and the placebo group. The between-group differences compared with placebo were −0.05388 (−0.6800 to 0.5723; Standard Error, SE = 0.3192; *p* = 0.8660) for the ω3-PUFA group, −0.3936 (−1.0217 to 0.2345; SE = 0.3180; *p* = 0.2192) for the multidomain intervention plus placebo group, and −0.6017 (−1.2255 to 0.02222; SE = 0.2092; *p* = 0.3202) for the combined intervention group. No significant effect was also found for the handgrip strength. Sensitivity analyses performed among participants with low (DHA+EPA) erythrocyte level at baseline (first quartile vs. others) or highly adherent participants (≥75% of the multidomain intervention sessions) revealed similar results. Conclusion: Low dose ω3-PUFA supplementation, either alone or in combination with a multidomain lifestyle intervention comprising physical activity counselling, had no significant effects on muscle strength over 3 years in elderly people.

## 1. Introduction

The omega 3 polyunsaturated fatty acid (ω3-PUFA), a nutrient with anti-inflammatory properties [1,2] and mainly consumed as eicosapentaenoic acid (EPA) and docosahexaenoic acid (DHA), has demonstrated significant mortality reduction from cardiovascular disease in a large prospective cohort study of older adults [3]. The anti-inflammatory effects of ω3-PUFA may also be beneficial to prevent the loss of muscle strength associated with aging [4]. ω3-PUFA supplementation is of great interest because it is simple to use, safe and low cost, which could facilitate its long-term implementation in the older adult population. Hence, confirmation of the effect of ω3-PUFA on muscle strength in older adults may have clinical and public health implications.

Growing clinical evidence from both cross-sectional and longitudinal observational studies also support the idea that low plasma ω3-PUFA level is associated with poor physical performance, such as slow gait speed in healthy older adults [5,6,7]. However, interventional studies in older adults remain scarce. Few randomized controlled trials, with different duration and doses, have previously investigated the effect of ω3-PUFA supplementation on physical performance [4,8,9,10]. They reported conflicting results, but the heterogeneous design of these clinical studies makes them difficult to compare. These studies are also limited by short duration (3 to 6 months), small samples size (up to 126 participants), and lack of ω3-PUFA blood level measurement at baseline that preclude giving the supplementation to older people who need it. Moreover, these clinical studies did not investigate the potential additional effect of multidomain intervention including physical activity counselling in older adults. The role of ω3-PUFA on physical performance under exercise training is not clearly based on previous research, but some investigations performed on young healthy adults suggest that ω3-PUFA improves neurological and muscle adaptation with training and reduces post-exercise fatigue [11,12].

Our team recently reported, in elderly adults with memory complaints in the Multidomain Alzheimer Preventive Trial (MAPT) study, that long-term omega 3 polyunsaturated fatty acid supplementation did not result in improvement in the short physical performance battery (SPPB) score, a multicomponent physical performance test combining the repeated chair stand test, balance test and gait speed [13]. In the present research, our aim was to investigate the effect of ω3-PUFA supplementation and a multidomain intervention (combining physical activity, cognitive training, and nutritional advice), alone or in combination, compared with placebo, on muscle strength. Our hypothesis was also that ω3-PUFA supplementation results in additional benefits in participants with a low docosahexaenoic acid (DHA)/eicosapentaenoic acid (EPA) erythrocyte level at baseline and in participants with high adherence to the multidomain intervention sessions.

## 2. Subjects and Methods

### 2.1. Population and Protocol

The design [14] and main results [13] of the MAPT study have been previously described in detail. Briefly, the MAPT study is a 3-year, multicenter, randomized, placebo-controlled trial with four parallel groups. The main outcome of the MAPT study was to investigate the effect of an intervention on cognitive function and no significant effects were reported [14]. Participants were non-demented, aged 70 years or older, and community-dwelling, older people, who had either spontaneously complained to their physician about their memory, limitations in one instrumental activity of daily living, and/or usual gait speed ≤0.8 m per second in the 4-m walk test. Exclusion criteria were a Mini Mental State Examination (MMSE) score lower than 24, diagnosed dementia, and current supplementation with ω3-PUFA at baseline. All participants signed informed consent. The study was approved by the French Ethical Committee located in Toulouse (CPP SOOM II). The trial was registered at a publicly accessible database under the registration number NCT00672685.

Participants were recruited in 13 memory clinics in France and Monaco between 30 May 2008 and 24 February 2011. Participants were randomly assigned (1:1:1:1) using a computer-generated randomization procedure to one of the four following groups: (I) ω3-PUFA alone (two capsules a day, providing a total daily dose of 800 mg DHA and 225 mg EPA), (II) ω3-PUFA plus the multidomain intervention (43 group sessions integrating advice for physical activity (PA), nutrition, cognitive training, and three preventive consultations), (III) the multidomain intervention plus placebo, or (IV) placebo alone. The concealment of group assignment was guaranteed.

The study staff and the participants were blinded to polyunsaturated fatty acid or placebo assignment. The capsules looked identical and tasted similar. Study staff involved in the clinical assessment of the participants, including the physical performance tests, were blinded to group assignment.

All participants took either two capsules of ω3-PUFA or two capsules of placebo. The ω3-PUFA supplement was V0137, and contained natural fish oil (400 mg DHA and 112.5 mg EPA). Placebo capsules contained flavored paraffin oil. Blood sample analysis of docosahexaenoic acid (DHA)/eicosapentaenoic acid (EPA) erythrocyte level was performed at baseline.

The multidomain intervention was focused on three domains: cognitive stimulation, physical activity counselling and nutritional advice. Each session was organized in group and lasted two hours. Two sessions per week were organized in the first month (8 sessions), and one session per week in the second month (4 sessions). Each session planning was standardized with 1 h of cognitive training, 45 min of advice about physical activity and 15 min of nutritional advice. The cognitive counselling was mainly focused on reasoning and memory training. The physical activity training included personalized proposals during individual interviews to help the participant to reach the physical activity guidelines of 30 min walking per day or more, 5 days a week. The nutritional advice was based on the French National Nutrition and Health Program guidelines (Programme National Nutrition Santé; PNNS). The initial two months of intervention were reinforced in the multidomain groups by a 1-h session each month for 3 years and two further 2-h sessions organized at 12 and 24 months. A preventive cardiovascular risk factor consultation was also organized at baseline, 12 months, and 24 months in a face-to-face consultation with a physician. The short form of the Minnesota Leisure Time Physical Activity Questionnaire was used to define sedentary (below 383 Kcals expended per week for men and below 270 for women) [15].

### 2.2. Primary Outcome

The primary outcome was changed from baseline to 36 months on muscle strength. 

Muscle strength was measured using the repeated chair stand test (for the lower extremities) and the handgrip strength (for the upper extremities), two standardized and widely used methods for the measurement of muscle strength in older adults.

For the repeated chair stand test, participants used a straight-backed chair, placed with its back against a wall. Participants were first asked to stand from a sitting position without using their arms. If they were able to perform the task, they were then asked to stand up and sit down five times, as quickly as possible with arms folded across their chests. The time to complete five stands was recorded and used for the present analyses.

Handgrip strength (HGS) was measured for the dominant hand with a hydraulic hand dynamometer (Jamar^®^ Hydraulic Hand Dynamometer; Sammons Preston, Bolingbrook, IL, USA) using standardized procedures [16]. Dynamometer was adjusted so that the participant feels comfortable. The participant was standing with his arm upright and the dynamometer close to his body. Three successive pressures were performed. The highest result was collected. HGS was expressed in kilograms. HGS has been reported to be a good marker of physical performances in community-dwelling older people [17].

### 2.3. Secondary Outcomes

Walking speed (performed on a 4-m course) and balance tests [18] were assessed as secondary outcomes. 

For the walking speed, participants were asked to walk at their usual pace over a 4-m course. Participants were instructed to stand with both feet touching the starting line and to start walking after a specific verbal command. Participants were allowed to use walking aids (cane, walker, or other walking aid) if necessary, but no assistance was provided by another person. Timing began when the command was given, and time in seconds needed to complete the entire distance was recorded. The faster of two walks was used for the present analysis.

For the balance test, participants were asked to hold three increasingly challenging standing positions for 10 s each: (1) a side-by-side position; (2) semi-tandem position (the heel of one foot beside the big toe of the other foot); (3) tandem position (the heel of one foot in front of and touching the toes of the other foot). The balance test score (in seconds) was given by the sum of seconds for which the positions were held (range 0–30). A decline in balance was defined as an aggravating score compared to the previous score.

The SPPB was based on the walking speed, repeated chair stands, and balance. A score ranging from 0 to 4 according to cut points based on time to perform each of the three tasks was assigned, where 4 indicated the best performers and 0 the worst performers. A summary score ranging from 0 (worst performers) to 12 (best performers) was calculated by adding walking speed, repeated chair stands and balance scores.

All clinical outcomes were assessed at baseline, and at 6, 12, 24, and 36 months.

The adherence of the participant to the multidomain intervention was collected every 6 months and defined as the percentage of the multidomain intervention sessions attended. For supplementation, adherence was assessed by counting the number of capsules returned by participants (or based on treatment dates if the number of capsules was missing). Participants were considered high adherents if they attended at least 75% of the multidomain group sessions. 

### 2.4. Statistical Analysis

Baseline characteristics were summarized as the mean ± SD for continuous variables and as frequencies and percentages for categorical variables. Chi-2 tests for qualitative variables and Fisher tests for quantitative variables were used to compare baseline characteristics between the 4 groups of intervention.

To study the effect of the interventions on the change in each continuous outcome (the repeated chair stand test, handgrip strength, 4-m walk test, balance test, and the SPPB) at 36 months, we used linear mixed models including baseline, and 6-, 12-, 24- and 36-month follow-up data. These linear mixed models included a center-specific random intercept (when this parameter was significant) to take into account the correlation intra center and a subject-specific random intercept and slopes to take into account the correlation intra subject. For each model we included the following fixed effects: intervention group, time, and interaction between intervention group and time. Time was treated as a continuous variable and we included the model time^2^ and time^3^ terms when the trajectory forms were not linear. For each model, we presented a means estimate and 95%CI and statistical significance was set at a *p* value < 0.05.

A discrete-time cox proportional hazard model was conducted for investigating the association of MAPT interventions on the incidence of balance tests (event = the decrease of at least 1 point). Participants who did not experience the event were censored at their follow-up length or participants with an event were followed up to the first event. Tests based on interaction with time were used to assert the proportional hazards assumption for the intervention group.

For each outcome, two subgroup analyses were performed according to baseline red blood cell DHA and EPA concentrations (participants with low (DHA+EPA) erythrocyte level at baseline (lowest quartile, ≤4.83%) were compared with others quartiles) and according to the level of adherence to the multidomain intervention and ω3-PUFA supplementation during the 36 months follow-up (participants with adherence ≥75% for both intervention were compared with others).

All *p* values are presented before and after adjustment for multiple comparisons with the Hochberg procedure to take into account that three intervention groups were compared with the placebo group in each model [19].

All analyses were performed using SAS software version 9.4 (SAS Institute Inc., Cary, NC, USA).

## 3. Results

A total of 1680 participants (75.34 years +/− 4.42) were randomized and 1679 contributed to the modified intention-to-treat analysis. Demographic characteristics and score in the physical performances are reported in Table 1. No statistical differences on demographic characteristics, repeated chair stand test, handgrip strength and the other physical performances were found between groups at baseline. The completion was similar in the four groups and reasons for discontinuation were not significantly different between groups at 36 months. 

Details about adherence to the intervention have been previously reported in detail [13]. In summary, adherence was approximately 85% in the ω3-PUFA group, and approximately 50% in the multidomain interventional group and the group combining ω3-PUFA and the multidomain intervention. At 12 months, we observed a statistically significant increase of 3·52% (SE 0.11) of the concentrations of DHA and EPA in red blood cells in the multidomain intervention plus polyunsaturated fatty acids and of 3.29% (0.11) in the polyunsaturated fatty acids alone groups, compared to the two groups receiving placebo (mean change −0.02% (0.11) in the placebo group and 0.06% (0.11) in the multidomain intervention plus placebo group) (*p* < 0.0001).

Table 2 reports the estimated 3-year within-group mean change in physical performance from baseline. A small decline in the grip strength was observed in all four groups during the 3-year follow-up. A decline in the balance test was observed 477 times (number of worsening score events among the 1674 participants). The incidence of decline in the balance test was 14.28% of participants per year (CI 95%, 13.00–15.56) during the 3-year follow-up. 

The estimated 3-year between-group mean difference for the repeated chair stand test and the handgrip strength from baseline is reported in Table 3. No statistical differences were found before and after adjustment. Analyses performed on the walking speed test, SPPB (Table 2), and balance test (Table 4) revealed similar not statistically significant differences between groups.

In subgroup analyses, the effects of the interventions did not differ between groups according to (DHA+EPA) erythrocyte level at baseline or adherence to physical activity sessions (Table 5, Table 6 and Table 7).

## 4. Discussion

Basic research supports that ω3-PUFA has an anabolic effect on muscle tissue via the improvement of insulin sensitivity [20], mitochondrial functioning [21], muscle protein synthesis [22], and reduced muscle protein breakdown [23]. However, our study does not support a beneficial effect of long-term ω3-PUFA supplementation on muscle strength in older adults. In this large randomized controlled trial, ω3-PUFA supplementation resulted in no significant effects on the repeated chair stand test or the handgrip strength over the 3-year follow-up in elderly people. Even participants with a low (DHA+EPA) erythrocyte level at baseline or participants with a high adherence to the multidomain session did not benefit from ω3-PUFA supplementation on muscle strength.

Strong evidence supports that physical activity is the most powerful intervention to prevent functional decline in older adults. However, the recommendations of physical exercise practice for older adults are difficult to achieve, especially in the long term and in frail older adults. Promising results have been reported with new drugs for treating sarcopenia [24] but further studies are needed to support their usage in routine care to prevent functional decline in older adults [24]. The future of these new drugs for the treatment of sarcopenia will also rely on their cost and safety in the long term, especially in frail older adults. In this context, ω3-PUFA supplementation has been suggested as an appealing option to prevent the progressive decline of physical performance observed in older adults.

To the best of our knowledge, only four randomized controlled trials have previously investigated the potential benefit of ω3-PUFA supplementation alone on physical performance in older adults. Smith et al. reported a 2.3 kg improvement in handgrip strength after six months of 4 g ω3-PUFA supplementation (1.86 g EPA and 1.5 g DHA) compared to corn oil placebo in 60 healthy 60- to 85-year-old men and women [4]. Logan et al. reported a 7% improvement in the Timed Up and Go test after three months of 5 g/day fish oil (2 g/day of EPA, 1 g/day of DHA) supplementation compared to olive oil in 24 healthy community-dwelling older women [10]. Hutchins-Wiese et al. also reported a statistically significant improvement in walking speed in 126 postmenopausal women after six months of supplementation with fish oil (1.2 g EPA and DHA per day) compared to olive oil [9]. Conversely, Krzymińska-Siemaszko et al. reported no statistically significant differences in the handgrip strength and Timed Up and Go tests among 53 elderly people with low lean mass randomized to 1.3 g of PUFA (660 mg EPA, 440 mg DHA, 200 mg other omega-3 fatty acids) plus 10 mg of vitamin E or 11 mg of vitamin E alone during 12 weeks, [8]. Based on these few studies, no clear association appears between higher doses of ω3-PUFA supplementation and better physical performance improvement. In the MAPT study, a daily dose of ω3-PUFA supplementation of 225 mg EPA and 800 mg DHA was fixed in agreement with the French nutritional recommendations for safety (Agence Française de Sécurité Sanitaire des Aliments) to avoid adverse events that may occur with over 2 g per day but also to exceed the daily recommended intake (250 mg DHA and 250 mg EPA) [25]. Despite limited evidence in the literature, it can be observed that all studies with over 1 g/d of EPA had positive findings on physical performance while all studies with less than 1 g/d of EPA had negative findings. We cannot exclude that a higher dose of ω3-PUFA such as 4 to 5 g/day [4,10] may have resulted in a clinically significant improvement of physical performances compared to placebo. Whether the increased mean DHA/EPA levels in blood in participant during the 3-year follow-up was associated with improved physical performances remains to be explored. None of the previous clinical trials performed in older adults investigated the potential synergistic effect of physical activity counselling in combination with ω3-PUFA supplementation. Physical activity is the cornerstone of the prevention of mobility disability in older adults, but the usual recommended volume of training is difficult to achieve in older adults and in the long term. Even though a previous study using MAPT data has shown the positive effects of the MAPT multidomain intervention on overall PA [26], this PA increasing was not enough to improve physical function; this suggests that the multidomain intervention, even among participants with a high adherence, was not strong enough to result in a significant benefit on physical performance. Moreover, the present study failed to demonstrate that ω3-PUFA would potentiate the effects of physical activity for determining physical function in older adults as suggested in exercise training studies in healthy younger adults [11,12] and middle-aged/younger older adults [27]. Whether exercise training (not only PA counselling), perhaps more oriented towards resistance training, would lead to improved physical performance in adults over 70 years remains to be determined.

Our intervention study is by far the most prolonged and dimensioned on ω3-PUFA supplementation and muscle strength. However, this study has limitations worth mentioning: the MAPT study was first designed to investigate the effect of a multidomain intervention and ω3-PUFA supplementation alone or in combination, on cognitive decline [13]. Muscle strength and physical performance were only a secondary endpoint. However, the strengths of the MAPT study are the unique large sample size and long-term randomized, controlled design and the combination of ω3-PUFA supplementation with lifestyle intervention.

In conclusion, this large clinical trial does not support the idea that low dose ω3-PUFA supplementation, either alone or in combination with a multidomain lifestyle intervention comprising PA counselling, improves muscle strength and physical performance over 3 years in elderly people, even in participants with a low ω3-PUFA level at baseline or high adherence to the multidomain intervention.

## Figures and Tables

**Table 1:** Demographic characteristics and score of physical performance tests at baseline.

	ω3-PUFA	ω3-PUFA + Multidomain Intervention	Multidomain Intervention + Placebo	Placebo	*p*
*N*	422	417	420	420	
Gender (F/M; %)	16.02/9.11	15.66/9.17	16.44/8.58	16.62/8.40	0.70
Age (yrs, mean, SD)	75.67 (4.65)	75.50 (4.46)	75.05 (4.19)	75.13 (4.36)	0.22
Weight (kg, mean, SD)	68.70 (13.22)	68.65 (13.56)	68.26 (12.62)	68.09 (12.53)	0.88
Body Mass Index (BMI) (kg/m^2^, mean, SD)	26.28 (4.08)	26.19 (4.29)	26.00 (4.00)	25.99 (3.91)	0.67
> Graduate high level (%)	7.06	6.94	7.91	7.12	0.26
Sedentary (physical activity (PA) Minnesotta scale, %) *	16.14	14.88	12.65	13.56	0.49
Mini-Mental State Examination (MMSE) (mean, SD)	28.07 (1.62)	28.08 (1.58)	28.04 (1.62)	28.09 (1.55)	0.97
**Muscle Strength**					
Repeated chair stand test (s, mean, SD)	11.96 (3.53)	11.85 (4.30)	11.96 (4.72)	11.59 (3.95)	0.22
Handgrip strength (kg, mean, SD)	27.33 (9.40)	27.95 (9.40)	27.50 (9.10)	27.21 (9.26)	0.68
**Other Physical Performances**					
4-m walking speed (m/s^−1^, mean, SD)	1.08 (0.26)	1.09 (0.26)	1.08 (0.26)	1.10 (0.26)	0.62
Short Physical Performance Battery (SPPB) (mean, SD)	10.53 (1.55)	10.50 (1.77)	10.64 (1.70)	10.66 (1.58)	0.16

BMI, Body Mass Index; MMSE, Mini-Mental State; SPPB, Short Physical Performance Battery; * Percentages were calculated on the basis of the number of participants for whom data were available for each criterion; SD, Standard Deviation; PA, physical activity.

**Table 2:** Estimated 3-year within-group mean change in physical performance from baseline (95% CI).

	ω3-PUFA (*n* = 422)	ω3-PUFA + Multidomain Intervention (*n* = 417)	Multidomain Intervention + Placebo (*n* = 420)	Placebo (*n* = 420)
Repeated chair stand test (s, mean, 95% CI)	0.499 (0.0545 to 0.943)	0.159 (−0.287 to 0.606)	−0.048 (−0.490 to 0.392)	0.552 (0.111 to 0.993)
Handgrip strength (kg, mean, 95% CI)	−3.532 (−4.216 to −2.849)	−3.968 (−4.645 to −3.290)	−4.0244 (−4.699 to−3.349)	−3.9158 (−4.587 to −3.244)
4-m walking speed (m/s^−1^, mean, 95% CI)	−0.093 (−0.120 to −0.065)	−0.084 (0.111 to −0.057)	−0.066 (−0.093 to −0.039)	−0.077 (−0.105 to −0.050)
SPPB (mean, 95% CI)	−0.3660 (−0.550 to −0.181)	−0.315 (−0.499 to −0.132)	−0.187 (−0.370 to −0.004)	−0.255 (−0.437 to −0.072)

SPPB, Short Physical Performance Battery; SD, Standard Deviation.

**Table 3:** Estimated 3-year between-group mean difference in change in physical performances from baseline (95% CI).

	ω3-PUFA vs. Placebo	Raw *p* Value	Adjusted *p* Value *	ω3-PUFA + Multidomain Intervention vs. Placebo	Raw *p* Value	Adjusted *p* Value *	Multidomain Intervention + Placebo vs. Placebo	Raw *p* Value	Adjusted *p* Value *
Repeated chair stand test (s, mean, 95% CI)	−0.053 (−0.680 to 0.572)	0.866	0.866	−0.393 (−1.021 to 0.234)	0.219	0.438	−0.601 (−1.225 to 0.022)	0.058	0.176
Handgrip strength (kg, mean, 95% CI)	0.382 (−0.575 to 1.341)	0.433	0.914	−0.05253 (−1.006 to 0.901)	0.914	0.914	−0.108 (−1.060 to 0.843)	0.823	0.914
4-m walking speed (m/s^−1^, mean, 95% CI)	−0.015 (−0.053 to 0.023)	0.435	0.737	−0.00658 (−0.045 to 0.031	0.737	0.737	0.011 (−0.026 to 0.049)	0.555	0.737
SPPB mean, 95% CI)	−0.110 (−0.370 to 0.149)	0.403	0.646	−0.06058 (−0.319 to 0.198)	0.646	0.646	0.067 (−0.191 to 0.325)	0.609	0.646

SPPB, Short Physical Performance Battery; * Adjusted for multiple comparisons with the Hochberg procedure to account for the fact that three intervention groups were compared with the placebo group in each analysis; SD, Standard Deviation.

**Table 4:** Estimated risk of 3-year balance performance decline (95% CI).

	N	Number of Events	Incidence (% Participant/y)	95% CI	RR *	95% CI	Raw *p* Value	Adjusted *p* Value
ω3-PUFA	420	119	14.63	12.00–17.26	1.03	0.79–1.35	0.834	0.834
ω3-PUFA + Multidomain Intervention	416	122	14.76	12.14–17.38	1.05	0.80–1.37	0.736	0.834
Multidomain Intervention + Placebo	419	116	13.74	11.24–16.24	0.95	0.73–1.25	0.720	0.834
Placebo	419	120	14.02	11.51–16.53	1	-	-	-

* Interaction group time; *p* = 0.4327.

**Table 5:** Estimated 3-year mean difference in change from baseline on repeated chair stand test in the three intervention groups compared with the control group (95% CI).

	ω3-PUFA vs. Placebo Mean (n; 95% CI)	Raw *p* Value (within Sub-Group Intervention vs. Placebo)	Adjusted *p* Value * (within Subgroup Intervention vs. Placebo)	ω3-PUFA + Multidomain Intervention vs. Placebo Mean (n; 95% CI)	Raw *p* Value (within Sub-Group Intervention vs. Placebo)	Adjusted *p* Value * (within Subgroup Intervention vs. Placebo)	Multidomain Intervention + Placebo vs. Placebo Mean (n; 95% CI)	Raw *p* Value (within Sub-Group Intervention vs. Placebo)	Adjusted *p* Value * (within Subgroup Intervention vs. Placebo)
Low DHA and EPA in red blood cells †	0.262 (96; −1.046 to 1.571)	0.694	0.694	0.270 (105; −1.046 to 1.587)	0.686	0.694	−0.429 (85; −1.794 to 0.935)	0.537	0.694
Normal DHA and EPA in red blood cells †	−0.093 (283; −0.836 to 0.648)	0.804	0.804	−0.482 (265; −1.225 to 0.260)	0.202	0.405	−0.5167 (290; −1.249 to 0.216)	0.166	0.405
High adherent to the Multidomain intervention (at least 75% of the sessions)	0.170 (330; −0.513 to 0.853)	0.625	0.625	−0.348 (333; −1.029 to 0.334)	0.317	0.625	−0.167 (348; −0.837 to 0.503)	0.625	0.625
Low adherent to the Multidomain intervention (less than 75% of the sessions)	−1.395 (65; −3.031 to 0.242)	0.094	0.189	−0.415 (57; −2.117 to 1.288)	0.633	0.633	−3.369 (52; −5.132 to −1.606)	<0.001	<0.001

BMI, Body Mass Index; MMSE, Mini-Mental State Examination; SPPB, Short Physical Performance Battery. A positive value of change is in favor of the intervention, whereas a negative value is in favor of the placebo. DHA = docosahexaenoic acid. EPA = eicosapentaenoic acid. * Adjusted for multiple comparisons with the Hochberg procedure to account for the fact that three intervention groups were compared with the placebo group in each analysis; † Low concentrations of DHA and EPA were defined by the lowest quartile of DHA plus EPA percentage at baseline in the modified intention-to-treat population (i.e., 4.83); SD, Standard Deviation.

**Table 6:** Estimated 3-year mean difference in change from baseline on handgrip strength in the three intervention groups compared with the control group (95% CI).

	ω3-PUFA vs. Placebo Mean (n; 95% CI)	Raw *p* Value (within Sub-Group Intervention vs. Placebo)	Adjusted *p* Value * (within Sub-Group Intervention vs. Placebo)	ω3-PUFA + Multidomain Intervention vs. Placebo Mean (n; 95% CI)	Raw *p* Value (within Sub-Group Intervention vs. Placebo)	Adjusted *p* Value * (within Sub-Group Intervention vs. Placebo)	Multidomain Intervention + Placebo vs. Placebo Mean (n; 95% CI)	Raw *p* Value (within Sub-Group Intervention vs. Placebo)	Adjusted *p* Value * (within Sub-Group Intervention vs. Placebo)
Low DHA and EPA in red blood cells †	0.423 (106; −1.564 to 2.410)	0.676	0.845	0.206 (101; −1.740 to 2.153)	0.835	0.845	−0.202 (86; −2.236 to 1.832)	0.8455	0.845
Normal DHA and EPA in red blood cells †	0.324 (287; −0.770 to 1.418	0.561	0.974	−0.018 (287; −1.112 to 1.076)	0.974	0.974	−0.137 (287; −1.216 to 0.942)	0.9740	0.803
High adherent to the Multidomain intervention (at least 75% of the sessions)	0.220 (335; −0.824 to 1.264)	0.425	0.447	−0.178 (59; −1.211 to 0.855)	0.447	0.447	−0.254 (354; −1.273 to 0.766)	0.3964	0.396
Low adherent to the Multidomain intervention (less than 75% of the sessions)	1.769 (65; −0.658 to 4.196)	0.152	0.458	0.669 (340; −1.830 to 3.167)	0.599	0.599	1.122 (53; −1.473 to 3.716)	0.3964	0.5997

BMI, Body Mass Index; MMSE, Mini-Mental State Examination; SPPB, Short Physical Performance Battery. A positive value of change is in favor of the intervention, whereas a negative value is in favor of the placebo. DHA = docosahexaenoic acid. EPA = eicosapentaenoic acid. * Adjusted for multiple comparisons with the Hochberg procedure to account for the fact that three intervention groups were compared with the placebo group in each analysis; † Low concentrations of DHA and EPA were defined by the lowest quartile of DHA plus EPA percentage at baseline in the modified intention-to-treat population (i.e., 4.83); SD, Standard Deviation.

**Table 7:** Estimated 3-year mean difference in change from baseline on total SPPB score in the three intervention groups compared with the control group (95% CI).

	ω3-PUFA vs. Placebo Mean (n; 95% CI)	Raw *p* Value (within Sub-Group Intervention vs. Placebo)	Adjusted *p* Value * (within Sub-Group Intervention vs. Placebo)	ω3-PUFA + Multidomain Intervention vs. Placebo Mean (n; 95% CI)	Raw *p* Value (within Sub-Group Intervention vs. Placebo)	Adjusted *p* Value * (within Sub-Group Intervention vs. Placebo)	Multidomain Intervention + Placebo vs. Placebo Mean (n; 95% CI)	Raw *p* Value (within Sub-Group Intervention vs. Placebo)	Adjusted *p* Value * (within Sub-Group Intervention vs. Placebo)
Low DHA and EPA in red blood cells †	0.129 (109; −0.411 to 0.670)	0.638	0.638	−0.139 (100; −0.674 to 0.396)	0.610	0.638	0.227 (87; −0.332 to 0.786)	0.425	0.638
Normal DHA and EPA in red blood cells †	−0.110 (285; −0.410 to 0.190	0.472	0.964	0.006 (190; −0.293 to 0.306)	0.964	0.964	−0.021 (304; −0.317 to 0.275)	0.888	0.964
High adherent to the Multidomain intervention (at least 75% of the sessions)	−0.110 (278; −0.382 to 0.161)	0.425	0.447	0.115 (201; −0.182 to 0.413)	0.447	0.447	0.178 (238; −0.109 to 0.466)	0.225	0.447
Low adherent to the Multidomain intervention (less than 75% of the sessions)	−0.284 (102; −0.781 to 0.211)	0.260	0.504	−0.249 (199; −0.583 to 0.085)	0.143	0.431	−0.118 (179; −0.466 to 0.229)	0.504	0.504

BMI, Body Mass Index; MMSE, Mini-Mental State Examination; SPPB, Short Physical Performance Battery. A positive value of change is in favor of the intervention, whereas a negative value is in favor of the placebo. DHA = docosahexaenoic acid. EPA = eicosapentaenoic acid. * Adjusted for multiple comparisons with the Hochberg procedure to account for the fact that three intervention groups were compared with the placebo group in each analysis; † Low concentrations of DHA and EPA were defined by the lowest quartile of DHA plus EPA percentage at baseline in the intention-to-treat population (i.e., 4.83); SD, Standard Deviation.
